# Energy diagram: Investigation and application of a design-thinking-driven wind environment simulation tool for sustainable architecture

**DOI:** 10.1371/journal.pone.0342247

**Published:** 2026-02-11

**Authors:** Wenzhou Zhong, Ke Li, Yongjie Pan, Yuan Yao, Haoran Wu, Wei Xiao, Tong Zhang

**Affiliations:** 1 School of Architecture, Southeast University, Nanjing, Jiangsu Province, China; 2 School of Environment and Society, Institute of Science Tokyo, Tokyo, Japan; 3 Department of Built Environment, National University of Singapore, Kent Ridge, Singapore; 4 School of Architecture, Nanjing Tech University, Nanjing, Jiangsu Province, China; 5 School of Civil Engineering, Jiangsu Open University, Nanjing, Jiangsu Province, China; Changan University: Chang'an University, CHINA

## Abstract

Amid climate change and resource constraints, sustainable building design increasingly requires wind-environment optimization to improve energy efficiency and thermal comfort. However, most simulation tools target late design stages, overlooking early phases where small geometric choices have an outsized performance impact. Through comparative software analysis and questionnaire survey, this study addresses the disconnect between designers’ workflows and existing tools, rooted in divergent thinking paradigms: designers’ design thinking and engineers’ scientific thinking. Accordingly, we propose “Energy Diagram,” a grey-box-based tool that integrates 2D Zonal models simplified by the Lattice–Boltzmann method with deep neural networks (DNNs) to predict wind fields by seamlessly coupling architectural diagrams with numerical simulations. Validation against wind-tunnel experiments, field measurements, and CFD simulations shows that, the mean MAPE of Energy Diagram is 16.85% (vs. experiments) and 10.45% (vs. simulations) for a cube case, and 19.21% (vs. measurements) and 13.79% (vs. simulations) for a reading-room case. Through application in an architectural studio, the characteristics of the tool, i.e., the visual integration, geometric transition, and human-machine collaboration, are verified and discussed. This research underscores the potential of human-centric tools to democratize performance simulation, empowering designers as proactive agents in sustainable architecture development.

## Introduction

### Background

Constructing sustainable buildings to shape habitable environments, given the context of climate change and resource scarcity, is self-evident [1]. The design of these buildings serves as a crucial juncture in determining their energy efficiency and carbon reduction capabilities [2,3]. Notably, the wind environment design and the resultant natural ventilation performance of buildings play a pivotal role in this process, particularly in enhancing thermal comfort [4,5], reducing indoor pollution levels [6], decreasing energy consumption of heating, ventilation, and air conditioning (HVAC) auxiliary systems [7,8], and mitigating carbon emissions [9]. Field observations [10], wind tunnel tests [11], and numerical simulations [12] constitute the three primary methods of investigation of the wind environment. Compared to these measurements and experiments, numerical simulations offer advantages such as convenience, high spatial resolution, and broad applicability [12–15].

Airflow modelling provides invaluable insights for understanding and controlling air currents in wind-driven architectural designs [16,17]. In recent years, the trend of utilizing wind environment simulation as one of the driving forces in sustainable building design has garnered significant attention. The interactive feedback loop between performance simulation and decision-making, facilitated by computational modelling, is emerging as a key strategy for optimizing a suite of related parametric variables in the design process.

### Literature review

**Fig 1:**
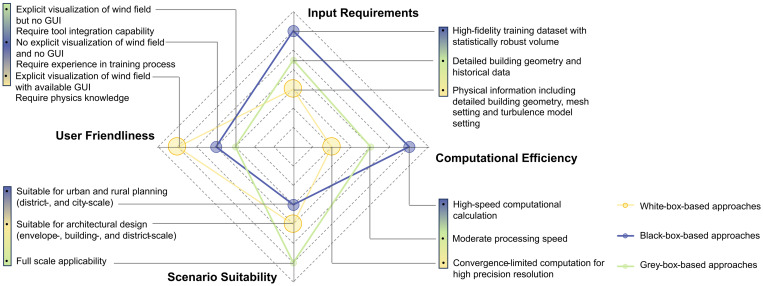
The advantages and disadvantages of white-box-based, black-box-based, and grey-box-based approaches of wind environment simulation.

To achieve this, a plethora of research cases employing state-of-the-art technologies and methodologies has come to prominence. These methods can be broadly categorized into three types: white-box-based, black-box-based, and grey-box-based approaches [16,18]. White-box methods, as traditional and widely used means for wind environment simulation, rely on a clear interpretation of the formulas derived from physical laws, typically involving detailed modelling of building geometries, boundary conditions, and fluid dynamics [19]. In contrast to white-box methods, black-box methods do not rely on detailed physical modelling but instead use statistical or machine learning algorithms to predict building wind environments [20] and ventilation efficiency [21], which have gradually become research hotspots in recent years. Grey-box methods represent a compromise between white-box and black-box approaches, combining simplified physical modelling and statistical prediction [22,23]. Each method possesses its unique strengths and weaknesses concerning computational efficiency, suitability for various scenarios, and user-friendliness (Fig 1). According to Zhang et al.’s review study [16], the majority of technological research and development endeavors, particularly those focusing on recent advancements in black-box and grey-box methodologies, are primarily directed towards scientific exploration led by experts like researchers and engineers. However, a significant proportion of designers, as non-experts in both physical formula derivation and code programming, continue to favor the utilization of white-box-based commercial software equipped with graphical user interfaces (GUI) [24].

**Fig 2:**
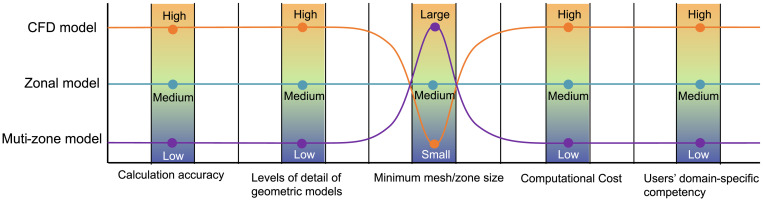
The advantages and disadvantages of the three distinct models of white-box-based approaches.

From designers’ perspective, the optional white-box-based airflow modelling approaches can be encapsulated within three distinct models: Multi-zone models, Zonal models, and Computational Fluid Dynamics (CFD) models [25]. The advantages and disadvantages of these three models are summarized in Fig 2. Ideally, designers can select the appropriate model based on the required level of accuracy, the problem to be solved, the affordable computation time and resources, as well as the users’ experience level [26].

**Fig 3:**
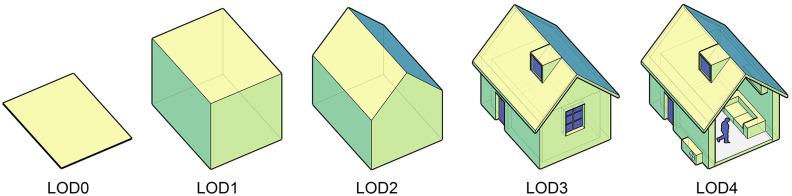
The five LODs of geometric models in simulation-driven wind environment design (the characteristic details of each LOD level are modified from Biljecki et al. [29]).

However, designers’ workflow and model selection often do not fully align in practical applications. During the wind environment design process for sustainable architecture, the associated geometry creation evolves through a meticulous refinement of the design scheme [27]. It begins with the foundational elements of the building’s plan and section, progresses to the determination of its volume and shape, and subsequently focuses on the placement of doors and windows. Ultimately, it comes to the final stages of operation and maintenance with the integration of furniture and equipment. Throughout this increasingly tangible process, fresh perspectives emerge to tackle intricate and undefined challenges [28]. Each stage of work aligns with geometric models that vary in levels of detail (LOD), which have been defined and visualized by Biljecki et al. [29] (Fig 3), necessitating the use of corresponding wind environment simulation tools [30]. However, the simulation tools applicable to each stage of the design process are unevenly distributed [31]. Most simulation software packages require clear geometric models, boundary conditions, and material parameters, essentially indicating that they can only be involved in the later stage of design with LOD3–4 [30,32]. However, performance simulation and design optimization in the early stage of design (LOD0–1) exert a greater effect on the final energy efficiency of buildings [33–35]; nonetheless, technical tools that can match the granularity for the early stages of design are relatively scarce [32].

As is made evident, there exists a disparity between the designers’ workflow and the software tools developed by the researchers and engineers. Most existing simulation tools fail to precisely match the granularity of geometric models or to reasonably quantify design elements during data input [30]. Furthermore, many designers, due to limited familiarity with physical models and simulation tools, frequently struggle to derive formal strategies directly from the output data [36]. As a result, designers often encounter obstacles when attempting to seamlessly integrate these tools into their workflows in practical projects.

### Contents and methods of this study

To summarize, the primary objective of this article is to bridge the gap between experts and non-experts, fostering collaboration that will ultimately drive the creation of comprehensive design and research tools. To identify research questions, we compared existing white-box-based commercial software commonly used in design. Additionally, a questionnaire survey was conducted targeting professional designers as well as faculty and students in schools of architecture. Ethical approval was obtained from the IEC for Clinical Research of Zhongda Hospital, Affiliated to Southeast University (Approval No.: 2023ZDKYSB187). Written informed consent was obtained from all respondents, who were fully informed about the purposes of this research and how their responses would be used and stored.

Through an analysis of the differences between designers’ design thinking and the scientific thinking that guides engineers and their simulation tools, the genuine needs of designers for simulation-driven wind environment design were identified. Accordingly, a grey-box-based simulation tool designed especially for LOD0 stage, called “Energy Diagram,” was proposed by coupling architectural diagram with computational simulation through model tessellation, algorithm simplification, and machine learning. The effectiveness of the simulation tool was verified and validated through empirical research. This research could hopefully contribute to the transformation of digital tools for sustainable building design and further promote possible innovation.

## Question formulation

### Comparison of the current design-used simulation tools

The frequently utilized wind environment simulation software is presented in Table 1, with a comparative analysis of their distinct characteristics, including model type, grid method, computational cost, LOD of applicable geometric models, the availability of GUI, the capability of wind field visualization, and the platform they are compatible with [16,24,37]. Several factors emerge as critical for design use:

**Table 1:** Comparison of different wind environment simulation software commonly applied in sustainable architectural design.

Tool	Model Type	Grid Method	Computational Cost	LOD of Geometric Model	GUI Available	Wind Field Visualization	Platform
OpenFOAM	CFD model	blockMesh snappyHexMesh	High	3-4	×	Require additional post-processing applications	Standalone
Ansys Fluent	CFD model	ANSYS Meshing ICEM CFD Mosaic	High	3-4	√	Available	Standalone
Phoenics	CFD model	Cube Mesh	Moderate to High	3-4	√	Available	Standalone
Butterfly	CFD model	blockMesh snappyHexMesh	Moderate	3	√	Require additional post-processing applications	Built in Rhino/Grasshopper
Envi-met	CFD model Zonal model	Cube Mesh	Moderate to High	3	√	Available	Standalone/plugin for Sketchup, Rhino and Revit
Winair	Zonal model	Grid Mesh	Low to Moderate	3	√	Require additional post-processing applications	plugin for Ecotect
SimScale	Zonal model	Finite-volume Mesh	Low	3	√	Available	Standalone
EnergyPlus /OpenStudio	Muti-zone model	Zone-based	Low	1-4	×/√	Unavailable	Standalone/plugin for Sketchup, Rhino and Revit
DesignBuilder	Muti-zone model	Zone-based	Low	1-4	√	Unavailable	Standalone
TRNSYS	Muti-zone model	Zone-based	Moderate	1-4	√	Unavailable	Standalone
eQUEST	Muti-zone model	Zone-based	Low to Moderate	1-4	√	Unavailable	Standalone/ plugin for AutoCAD-based software

ⅰ. In terms of model types, pieces of software based on CFD models (such as OpenFOAM, Ansys Fluent, Phoenics, and Butterfly) generally require intricate mesh processing [38]. Some tools necessitate additional specialized external applications solely for mesh generation. Furthermore, their calculations demand more detailed geometric models (LOD ≥ 3), leading to higher computational costs. However, their advantage lies in providing visualizable computational results of wind fields, either inherently or through supplementary post-processing applications. On the other hand, software based on Zonal models (like Envi-met and Winair), due to their use of relatively coarse meshes, can offer acceptable visual representations of wind fields while reducing computational load. As for software rooted in Multi-zone models (such as DesignBuilder, TRNSYS, and eQUEST), they are primarily used for calculating building energy consumption. These tools treat individual rooms as a uniform airflow and temperature fields, utilizing air changes per hour (ACH) to characterize ventilation performance. Consequently, they cannot provide visual wind fields. Their strength is minimal computational costs and adaptability to geometric models of varying levels of detail (LOD1–4).
ⅱ. From the perspective of LOD applicability in geometric models, models requiring higher precision in solving wind environments demand a correspondingly higher LOD of geometric models. This is particularly evident in the simulations of natural ventilation, where it is essential to establish LOD3 models coupling enclosed structures that distinguish between indoor and outdoor boundaries, with openings that connect the interior and exterior. For even more refined simulations of indoor wind fields with HVAC systems, additional consideration must be given to more intricate geometric models of equipment, people, and other facilities (LOD4). In contrast, software primarily focused on energy consumption simulation rather than CFD calculations can support geometric models with a broader range of LODs. Conversely, commercial software intended for the LOD0 stage, which corresponds to the early stages of architectural design such as plan and section sketches, is virtually nonexistent.
ⅲ. From a user’s perspective, apart from the complexity of using the software itself and the difficulty of acquiring relevant knowledge, post-processing requirements, GUI usability, and platform compatibility are three additional factors that may influence users’ choices. A detailed discussion on these aspects is provided in the next section.

### Questionnaire survey: Designer’s preferences and needs

A questionnaire (see supplementary materials) was designed to gather insights from practitioners, teachers, and students regarding their experiences, habits, and feelings toward the use of wind environment simulation software in sustainable architecture design.

To maximize the validity of the collected data, particularly in terms of its potential causal relationships with designers’ workflow and tools utilized, it is crucial to ensure that respondents provide answers based on their experiences in design practice. The questionnaire was distributed online from September 1st to December 16th, 2024. The questionnaire was anonymous, and the authors had no access to information that could identify individual participants during or after data collection. Considering that the target users for the tool are professionals in China’s construction industry, the questionnaire was targeted at domestic users. A total of 537 questionnaires were completed, with 504 deemed valid, resulting in a response rate of 93.9%. Among the 504 respondents, there were 293 practitioners and 211 students. Respondents from architectural design, urban and rural planning, landscape design, and environmental engineering accounted for 32.54%, 23.41%, 16.67%, and 27.38% respectively. Software-choice reasons by industry sector and professional status are shown in Table 2.

**Table 2:** Cross-tabulation of industry sector and professional status by software-choice reasons.

Industry sector	Professional status	Software-choice reasons				
		Easy to use	High efficiency	Good visualization	High accuracy	Good platform compatibility
Architectural Design	Practitioner	54	55	44	32	36
	Student	31	31	31	24	17
Urban and Rural Planning	Practitioner	41	33	34	23	19
	Student	33	24	26	20	16
Landscape Design	Practitioner	21	24	21	21	18
	Student	21	20	24	16	13
Environmental Engineering	Practitioner	41	44	32	31	17
	Student	30	31	28	21	15

**Fig 4:**
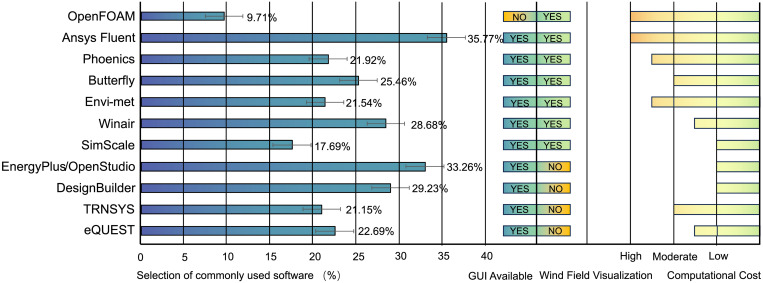
Vote for the most used software obtained from the questionnaire survey.

**Fig 5:**
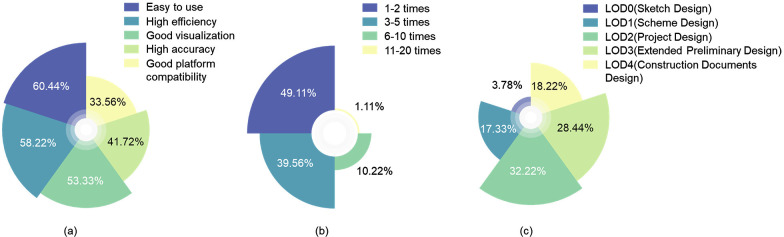
Data obtained from the questionnaire survey: (a) What are the main reasons for your choice of current software (Multiple choices allowed); (b) What is the average number of cycles of wind environment simulation and design interaction feedback you conduct in a project; (c) At which stage of design do you most frequently perform wind environment simulation.

Several findings can be identified from the questionnaire survey:

ⅰ. A significant 89.29% of respondents have utilized wind environment simulation software in the design process, indicating a substantial market demand for such tools.
ⅱ. The most frequently used software, as voted by participants, is listed in Fig 4. Ease of use, high efficiency, and visualization have emerged as the primary criteria for designers when choosing software tools, garnering vote percentages of 60.44%, 58.22%, and 53.33%, respectively, surpassing those for accuracy and platform compatibility (Fig 5a).
ⅲ. The average number of cycles of simulation and design interaction conducted within a project is illustrated in Fig 5b, with an average of 3.3 iterations. In contrast, Flager et al.‘s research [39] reported 2.7, indicating that the interactive feedback loop between performance simulation and decision-making facilitated by computational modelling is constrained by various factors, resulting in an insufficient number of cycles to identify the optimal performance solution.
ⅳ. The voting results for the design stages during which wind environment simulations are most frequently conducted are summarized in Fig 5c. These five stages can broadly correspond to the five LOD levels of the geometric models, with the Project Design stage receiving the highest proportion at 32.22%, while the Sketch Design stage received the least at only 3.78%. Meanwhile, 62.10% of the respondents expressed a desire to conduct simulations during the Sketch Design stage, indicating a mismatch between the demand for tools and their availability at this early stage of design.

### Question formulation: From designer’s perspective

Overall, the realistic problem of simulation-driven wind environment design is embodied, at the surface-layer requirements, in the lack of designer-friendly simulation tools applicable to the early stages of the design process [30,40], and at the bottom-layer logic, is manifested in the fundamental differences in the thinking paradigms followed by designers and engineers [36,41]. Recognizing and understanding such differences will be of considerable research value and realistic significance.

## Position of thinking paradigm differences

Designers and engineers, as non-experts and experts in simulation software respectively, follow different thinking modes, method systems, and disciplinary paradigms [36,42]. However, frequent interactions are compulsory between design processes and simulation tools in the implementation of designers’ work, which, in essence, establishes a binary dialogue structure.

### Form versus energy

Designers focus on “form.” The operations and presentations of form depend on visual media [36], which was confirmed by the questionnaire survey. “Geometry” has become a carrier for organizing formal language and graphical representation. Design thinking highlights visual ability, and thus, the tools designers typically use cater to the purpose of operating visualized graphic primitives and presenting design information.

Engineers focus on the “performance.” Through “energy” represented by heat flow and temperature difference, the performance of buildings is parameterized into nonlinear partial differential equations with specific boundary conditions [43,44]. Engineers’ scientific thinking regards buildings as models of heat flow balance, which can be calculated, analyzed, and predicted by numerical simulation tools.

### Situation versus model

Designers act as “comprehensivists” in design practice. Establishing comprehensive overall solutions is necessary, because each project has its respective complexity and is often lacking clear assumptions, mathematical description, let alone clear goals [45]. Compared with “modelling,” designers typically construct a “situation” and establish “observation–operation–evaluation” cycles through continuous adjustment and feedback [36,46]. In this process, new insights are triggered, accompanied by the continuous refreshing and refining of design problems until a solution that satisfies the comprehensive design goal is found [28].

Engineers’ intellectual activity resembles that of a scientist, i.e., conducting scientific experiments through certain steps, such as assumption creation, modelling, initial condition setting, testing and calculation, and result verification [47,48]. The typical strategy of scientific experiments is variable isolation, and design optimization driven by numerical simulation via sensitivity analysis, parameter elimination, factorial analysis, and the Monte Carlo method essentially establishes the quantitative causal relationship between design parameters (input) and building performance (output) to guide the design decision [35,49].

### Structure of binary dialogue

Based on the preceding discussion, it becomes evident that the practical challenges encountered by designers when utilizing simulation tools in wind environment design, as emphasized in the questionnaire survey, stem from the disparate thinking paradigms adopted by non-experts and experts [36]. Therefore, establishing an efficient dialogue mechanism between them and developing a simulation tool that bridges the gap can not only respond to the realistic problem in sustainable architectural design through numerical simulation, but also facilitate the transformation of design tools under digitalization technologies.

### Simulation tool: “Energy Diagram”

Accordingly, we developed a design-thinking-driven wind environment simulation tool, called “Energy Diagram,” to establish a mapping between architectural graphic primitives and data parameters. Through it, the physical changes expressed by energy flow and environmental changes expressed by form can be mutually mapped and presented in a concise and efficient manner that fits design thinking. The “Energy Diagram” tool seamlessly integrates architectural diagrams with computational simulations by employing a 2D Zonal model and grey-box-based methodology. This integration is designed to strike an optimal balance between usability, efficiency, visualization, and accuracy—meeting the simulation tool requirements of designers at early design stages.

### Software development approach

**Fig 6:**
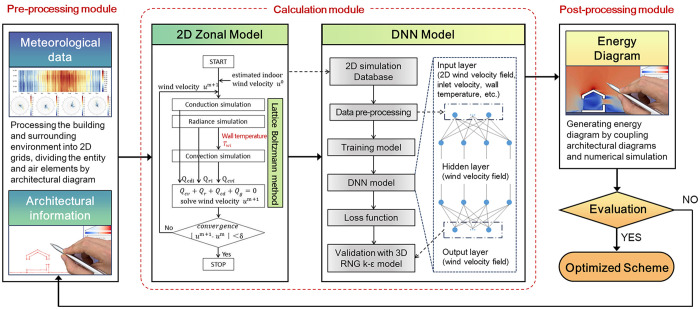
The proposed architecture for the “Energy Diagram” tool for predicting wind velocity field.

The “Energy Diagram” tool is divided into three software architecture layers, namely, the preprocessing module for graphic recognition, the calculation module for numerical simulation, and the post-processing module for data visualization, forming an “input–calculation–output” basic processing flow and system organization (Fig 6). During the tool coding stage, in accordance with the design requirements of the data structure, algorithm analysis and module implementation, we implemented the tool in Java and compiled it into an automated, serial-processing pipeline; the major algorithms, data structures, hierarchical organization, and inter-module call relations were organized to meet the tool’s functional, performance, and interface requirements.

The preprocessing module is highly integrated with the post-processing module into a built-in design platform, and the operating features of the design sketch drawing are restored via interactive interfaces. By processing the building and surrounding environment into 2D space grids, plans or sections of the building are drawn on the grid. The wall is defined by entity elements, while other grids are treated as independent finite air elements. The input of architectural information is based on the logic of “entity” and “air” on 2D grids, and thus, the computer-aided design platform or SketchUp platform based on 3D vector primitives was not chosen. Despite the resulting loss of compatibility with certain software, this processing method not only simplifies the calculation model, so that it can cater to the granularity of the early-stage design scheme, but also enhances overall work efficiency.

The calculation module is based on a 2D Zonal model that models building entities and air from plan and section sketches. The entity and air in the built environment are the constituent elements that serve as each other’s boundary conditions, and the numerical simulation of one party must be based on the other party’s definite value as the known condition. Hence, the governing equations for the air and the heat balance equation of the entity surface should be coupled in the data transfer process of the “Energy Diagram” tool, where the building’s wall temperature $T_{wi}\;$is used as the coupling variable to link the two equations in an iterative solution (Fig 6).

1) **Governing equations for the air**

Continuity equation:


∇·(u)=0 (1)

Momentum equation:


$$\frac{\partial (u)}{\partial t}+\nabla \cdot (uu)=-\nabla p_{rgh}-(g\cdot r)\nabla \left(\frac{\rho }{\rho_{\text{0}}}\right)+\nabla \cdot \left(\text{2}v_{eff}D(u)\right)$$ (2)

Temperature equation:


$$\frac{\partial (T)}{\partial t}+\nabla \cdot (Tu)=k_{eff}\nabla^{\text{2}}T$$ (3)

*u* is the velocity field, *p* is the pressure field, ρ is the density field, and g is the gravitational acceleration. $v_{eff}$ is the effective kinematic viscosity, and $k_{eff}$ is the effective thermal conductivity of air.

2) **Heat balance equation of the entity surface**

$$Q_{cv}+Q_{r}+Q_{cd}+Q_{g}=\text{0}$$ (4)

$Q_{cv}$represents convective heat transfer, $Q_{r}$ denotes radiative heat transfer, $Q_{cd}$ refers to conductive heat transfer, and $Q_{g}$ is the generation or dissipation term (e.g., heat gain from solar radiation or heat loss due to latent heat of evaporation).

The key step of the “Energy Diagram” tool to realize 2D coupled simulation by combining the above equations lies in the simplification based on the Lattice–Boltzmann method (LBM). The principle of this algorithm is described as follows. Fluid is simplified into microscopic particles, and the collision process of the microscopic fluid particles in lattice space is simulated by establishing discrete lattice grids and distribution functions, thereby describing the macroscopic behavior of the fluid [50,51]. This simplification process is described via the Boltzmann transport equation:


$$\frac{\partial f}{\partial t}+\vec{u}\cdot \nabla f=\;\Omega$$ (5)

where $f(\vec{x},t)$ represents the particle distribution function, $\vec{u}$ is the vector velocity of the particles, and Ω stands for the collision operator.

However, 2D simulation presents inherent limitations in accurately capturing wind performance within 3D environments [52]. To address this challenge, our study proposes a data-driven predictive framework that integrates machine learning (ML) techniques to enhance wind velocity field optimization. The DNN captures nonlinear mappings between environmental variables and velocity responses (Fig 6). Compared to conventional CFD methods, DNNs show strong capability in constructing intricate mapping functions from input parameters to output predictions through their multi-layered framework [53–55]. While direct application of ML approaches typically requires extensive training data for complex input conditions like building morphology [22,56], our hybrid methodology synergizes white-box simulations with DNN modeling. This integration achieves dual objectives: (1) maintaining prediction accuracy for wind velocity distributions, and (2) significantly reducing computational time and training costs. The resulting framework enables efficient parametric analysis, time-sensitive optimization processes, and diverse engineering applications.

A feedforward DNN model comprises an input layer, several hidden layers, and an output layer. Each layer contains one or more neurons, which establish connections with neurons in both preceding and subsequent layers [54,57]. The output of a neuron is typically computed using Eq. (6).


$$\hat{y}=f(\sum_{i=\text{1}}^{n}W_{i}x_{i}+b)$$ (6)

where $x_{i}$ denotes the *i*-th input to the neuron (which corresponds to the output of a neuron from the preceding layer), $W_{i}$ signifies the weight connecting the two neurons, *b* represents the bias of the neuron, and *f* stands for the activation function.

**Fig 7:**
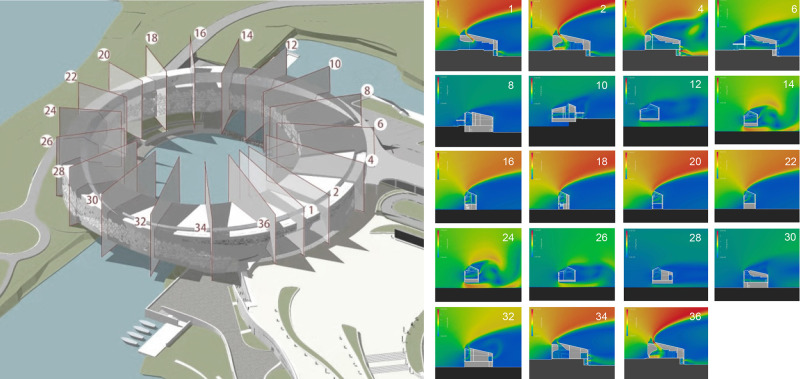
The 3D CFD simulations of a circular building with its cross-sectional slices serving as database.

The establishment of a DNN model involves a meticulous training and testing process. We built a database from 3D CFD simulations employing the RNG k–ε model of a cylindrical building with continuously varying cross-sections and openings. Each cross-sectional slice constitutes a sample with a corresponding 3D target velocity field (Fig 7). The database was then divided into two sets, namely, the training dataset (24 samples) and the test dataset (56 samples). The resulting train-test spilt was 30:70.

The DNN model was trained on the training dataset. Upon completion of the training phase, the input layer encompassed crucial parameters: the wind velocity field derived from the 2D Zonal modeling, inlet velocity, and wall temperature. The DNN architecture was a fully connected multilayer perceptron with the following specification: 8 hidden layers each with 64 neurons on each hidden layer. The activation function in hidden layers was ReLU. We optimized the network using Adam with a learning rate of 0.001. Batch size was 1–8 depending on case size to accommodate variable input tensor sizes while preserving stability on small cases, and the model trained for up to 1000 epochs. We used early stopping (patience = 4 epochs) and ReduceLROnPlateau (factor = 0.1, patience = 4) to mitigate overfitting. The Grid Search algorithm was applied, and Table 3 lists the optimal hyperparameters. Predictions of the wind velocity field were generated using the DNN model and subsequently validated by comparing them with the test dataset.

**Table 3:** Optimal hyperparameters.

Hidden layers × neurons	Activation function	Optimizer	Batch size	Training epochs	Loss function
8 × 64	ReLU	Adam	1-8	1000	MSE

To evaluate the model predictions against the actual output, three fitness metrics were employed: the coefficient of determination (R^2^), the root mean square error (RMSE), and the mean absolute error (MAE). Utilizing these metrics, and given the specific input features and desired output, the architecture of the DNN model underwent iterative reconfiguration to achieve the best prediction results.


$$R^{\text{2}}=\text{1}-\left[\frac{\sum_{i=\text{1}}^{n}{\left(\hat{y_{i}}-y_{i}\right)}^{\text{2}}}{\sum_{i=\text{1}}^{n}{\left(\overset{―}{y_{i}}-y_{i}\right)}^{\text{2}}}\right]$$ (7)

$$RMSE=\sqrt{\frac{\sum_{i=\text{1}}^{n}{\left(\hat{y_{i}}-y_{i}\right)}^{\text{2}}}{n}}$$ (8)

$$MAE=\frac{\text{1}}{n}\sum_{i=\text{1}}^{n}\left|\hat{y_{i}}-y_{i}\right|$$ (9)

The predictions generated by the model are denoted as $\hat{y_{i}}$, whereas the actual output is represented by *y*. Additionally, $\overset{―}{y}$ stands for the mean of the outputs.

The trained DNN maps 2D inputs to 3D-consistent velocity fields with substantially reduced computation time, enabling real-time visualization within the “Energy Diagram” tool. Users can interrogate single-cell values or export global fields for downstream analysis, supporting rapid design iterations and engineering applications.

### Workflow

**Fig 8:**
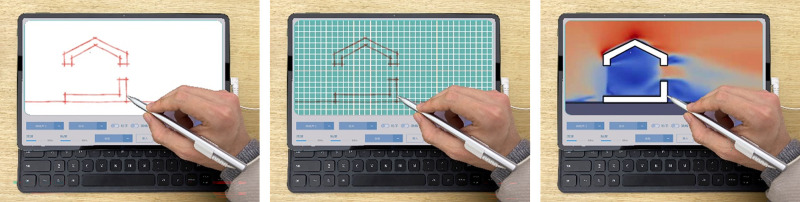
The operating mode and processing flow of the “Energy Diagram” tool.

The architectural design process driven by the “Energy Diagram” tool follows the order of “drawing–simulation–optimization (Fig 8),” which essentially simplifies the process of transforming the “design scheme” into a “parametric model” and implementing “performance simulation” in the traditional simulation-driven design workflow. On the one hand, the analysis of the simulation results can intuitively assess the uniformity of the wind field through visualization. On the other hand, the simulation results can be translated into formal strategies based on the closeness of quantitative performance indexes to the target value (Table 4).

**Table 4:** The criteria for determining the ideal range of building performance indicators.

Index		Indicator range	
Indoor average temperature $T_{in}$	$\text{0}.\text{4}\leq \frac{\left(T_{wi}-T_{in}\right)}{{(T}_{in}-T_{out})}\leq \text{0}.\text{8}$	(10)	$T_{wi}$–internal wall temperature, °C $T_{in}$–indoor average temperature, °C $T_{out}$–outdoor average temperature, °C
Indoor average wind velocity $V_{in}$	$\frac{\left(V_{out}-V_{in}\right)}{V_{out}}\leq \text{0}.\text{6}$	(11)	$V_{out}$–outdoor average wind velocity, m/s $V_{in}$–indoor average wind velocity, m/s
Indoor wind field uniformity *P*	$P=\frac{\left(V_{min/max}-V_{in}\right)}{V_{in}}\leq \text{0}.\text{5}$	(12)	${\text{V}}_{\text{min}/\text{max}}$–outdoor characteristic wind velocity, m/s $V_{in}$–indoor average wind velocity, m/s
Indoor wind field structure *H*	$H=\frac{S_{e}}{S_{\text{0}}}$	(13)	$S_{e}$–eddy current area, m^2^ $S_{\text{0}}$–indoor plane or section area, m^2^

### Validation study

Although performance simulations in early design phases do not require high absolute precision, validating the accuracy of the “Energy Diagram” tool remains critical. We therefore benchmarked the tool against two sets of experiments: (1) wind-tunnel tests of an isolated cube reported by Nakamura et al. [58] and the corresponding CFD simulations using three Reynolds-averaged Navier–Stokes (RANS) models—standard k–ɛ, RNG k–ɛ, and SST k–ω—combined with two near-wall treatments: wall functions (WF) and low Reynolds number modeling (LRNM), and (2) field measurements of a campus reading room and the corresponding CFD simulations using the RNG k–ɛ model with LRNM. Thermal boundary conditions were provided by EnergyPlus, and the flow field was computed in OpenFOAM.

**Fig 9:**
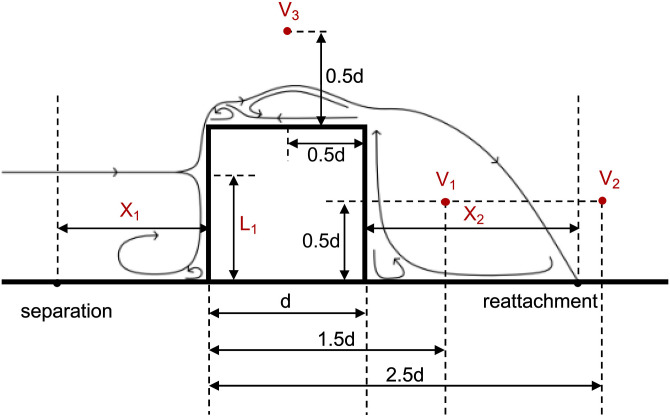
Indications of wind field and measurement in the wind-tunnel cube cases.

**Fig 10:**
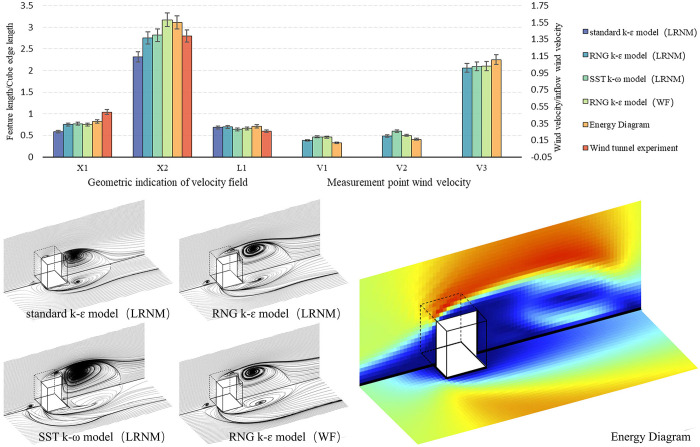
Verification of experiment data and numerical simulations in the wind-tunnel cube cases.

**Fig 11:**
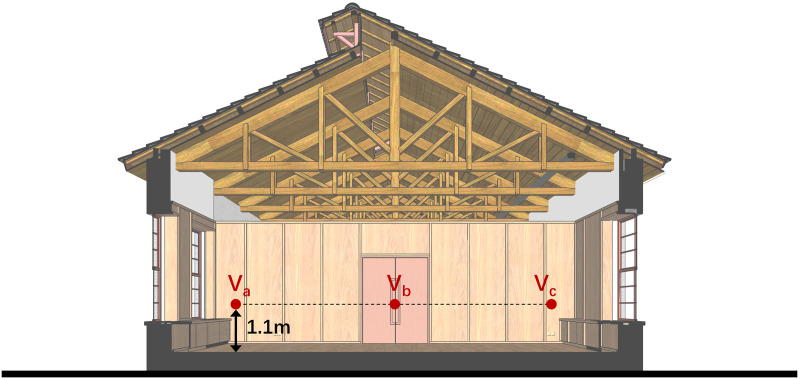
Probe points in the campus reading room measurement cases.

**Fig 12:**
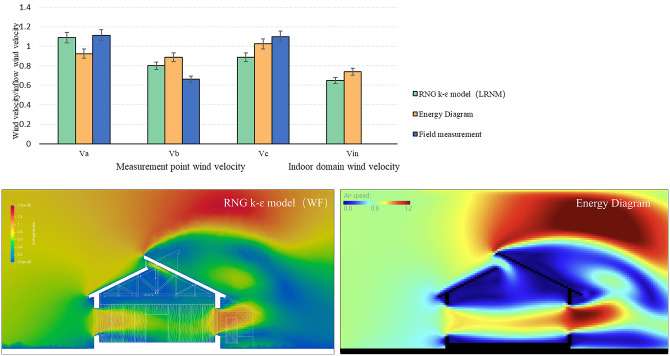
Verification of experiment data and numerical simulations in the campus reading room measurement cases.


Validation accuracy was evaluated using the Mean Absolute Percentage Error (MAPE) at co-located sampling points. In the wind tunnel cube cases, where reference measurements are limited, the sampling points were chosen based on the characteristic features of the wind field structure, along with wind velocities recorded at three probe points located around the cube (Figs 9–10). For the field measurements, three probe points in the reading room served as the measured sample, while the entire indoor velocity field was utilized as the simulated sample (Figs 11–12).

$$MAPE=\frac{\text{1}\text{0}\text{0}\%}{n}\sum_{i=\text{1}}^{n}\left|\frac{\hat{y_{i}}-y_{i}}{y_{i}}\right|$$ (14)

To avoid division instabilities at near-zero reference velocities, points with $\left|y_{i}\right|<\text{0}.\text{0}\text{5}$ were excluded from the percentage-error aggregation. Uncertainty was quantified via non-parametric bootstrap resampling of per-point absolute percentage errors. Case-wise MAPEs are summarized in Table 5.

**Table 5:** Case-wise validation errors.

Case	Reference	Validation target	MAPE (%) of Energy Diagram
Cube	Experiment	Wind tunnel experiment	16.85%
	CFD	standard k–ɛ model (LRNM)	26.3%
	CFD	RNG k–ɛ model (LRNM)	10.45%
	CFD	SST k–ω model (LRNM)	15.94%
	CFD	RNG k–ɛ model (WF)	11.9%
Campus reading room	Measurement	Field measurement	19.21%
	CFD	RNG k–ɛ model (LRNM)	13.79% (95% CI: 11.71–15.37%)

Through comparative analysis, several modelling parameters (e.g., wall temperature, grid density, inflow Reynolds number, near-wall approach, and turbulence model) in the simulation process of the “Energy Diagram” tool were determined as factors that influence accuracy. Subsequently, the Morris one-factor-at-a-time sensitivity analysis method was employed to vary the values of these parameters, with the objective defined as the absolute percentage error at co-located points to align with MAPE. Random sampling was conducted on the canonical cube cases with at least 15 trajectories, and the elementary effects were used to ascertain parameter sensitivity and select calibrated settings.

Under the above protocol, after calibration, the mean MAPE of the “Energy Diagram” tool was 16.85% (measured) and 10.45% (simulated) in the cube case; 19.21% (measured) and 13.79% (simulated) in the reading room case. As a reference, Gan et al. developed a physics-guided, data-driven (grey-box) surrogate to predict indoor ventilation (ACH) from CFD-derived façade pressures combined with multizone modeling, and reported that a fusion DNN achieved an absolute percentage error of 16.9% on a 25-sample test set relative to the multizone simulations. These errors are consistent with early-stage design needs, while balancing simulation accuracy and efficiency.

## Application feedback

To evaluate the applicability of the “Energy Diagram” tool, this study conducted an empirical investigation through a pedagogical case study in the senior design studio of a school of architecture at a leading Chinese university. The studio program, titled *Climate Configuration and Cultural Remodeling: Southern Yangtze Village Station*, challenged participants to design climate-responsive infrastructure for a lakeside village in Suzhou’s historic region. The pedagogical framework emphasized developing students’ competencies in architectural climate adaptation strategies, specifically training them to mitigate site-specific environmental conditions (e.g., solar exposure, wind patterns, and hydrological features) to minimize building cooling/heating demands through sustainable design interventions.

### Course organization

**Fig 13:**
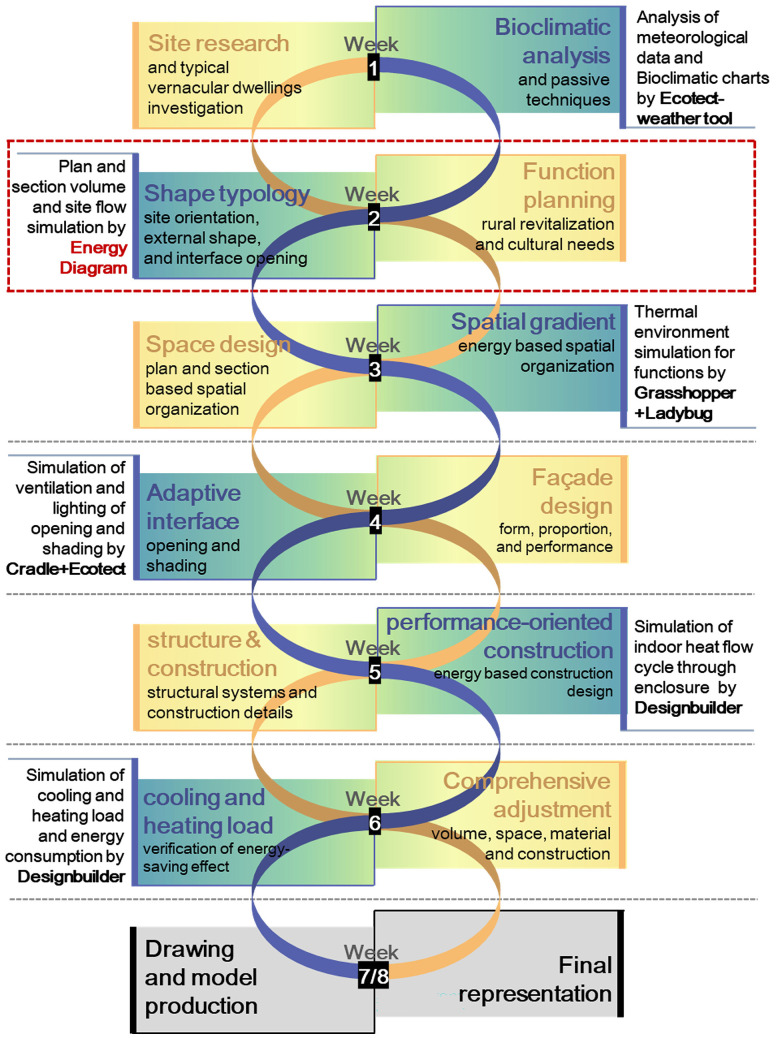
The intervention nodes of the “Energy Diagram” tool in the course structure.

The 8-week pedagogical framework was structured through dual interactive trajectories: spatial formation and performance analytics (Fig 13). The spatial design progression followed conventional architectural protocols spanning site research, function planning, space design, façade design, structural integration, and construction detailing. Concurrently, the performance evaluation framework incorporated bioclimatic analysis, shape typology assessments, spatial gradient analysis, adaptive interface optimizations, performance-oriented construction strategies, and comprehensive thermal load evaluations.

These dual trajectories maintained a dynamic, iterative relationship throughout the curriculum, with continuous feedback loops enabling mutual reinforcement. During Week 2, the instructional sequence integrated the “Energy Diagram”, aligning with the overall function planning through the preliminary simulation of form layout and site airflow. In this process, fundamental design determinants, such as space type selection, site orientation, external shape, and interface openings, were largely established.

### Tool application

**Fig 14:**
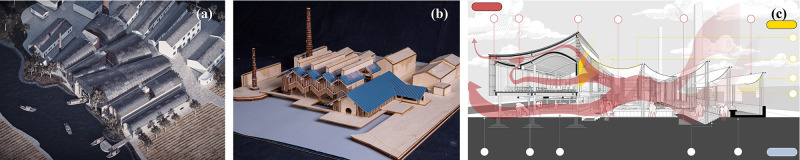
The final architectural schemes designed by the students: (a) Dock Museum, (b) Mahjong Parlor, and (c) Water Market.

Three student groups developed three architectural schemes in this course as application examples of the “Energy Diagram” tool, namely: (I) Dock Museum, (II) Mahjong Parlor, and (III) Water Market (Fig 14). Detailed information on the cases, such as function planning, performance targets, design strategy, simulation validation and optimization, and final outcome and innovation, is listed in Table 6.

**Table 6:** Detailed information of the 3 application cases of the “Energy Diagram” tool.

	Case I: Dock Museum	Case II: Mahjong Parlor	Case III: Water Market
Function planning	Exhibition and display of intangible cultural heritage, namely, the “Jinxi wooden boat”	Parlor for playing mahjong to activate communication and realize cultural inheritance	Agricultural product market that integrates sales and exhibition and caters to local villagers and tourists
Performance target	Optimize natural ventilation and wind heat environment to ensure thermal comfort and reduce HVAC load		
Design strategy	-Vault reduces turbulence and wind shadow -Parallel walls guide summer wind and block winter wind -Patio to enhance cross ventilation	-Referenced “roof-patio” ventilation strategy from Southern Yangtze vernacular dwellings to enhance natural wind-induced ventilation -Solar-assisted chimneys to enhance buoyancy-driven ventilation	-Referenced “air chipper” canopy domes from Singapore South Beach Complex -L-shaped block to block winter northwest wind -Double-layer curved roofs to enhance pressure difference in summer
Simulation validation & optimization by Energy Diagram	-Impact of courtyard width and building height on ventilation -Plan layout rotation and dislocation simulation -Interface opening and wind field uniformity validation	-Patio and pitched roof combination simulation -Optimization of solar chimney height, form, and bottom air inlets	-Validation of “air chipper” effectiveness -Optimization of L-shaped block cutting and stilt construction
Final outcome & innovation	-Three patios and narrow lanes between parallel walls form an effective natural ventilation system	-Achieved summer thermal comfort using patios, water-facing openings, and four rows of solar chimneys	-Integrated L-shaped block with canopies, using double-layer curved roofs to “shovel” airflow into the canteen

**Fig 15:**
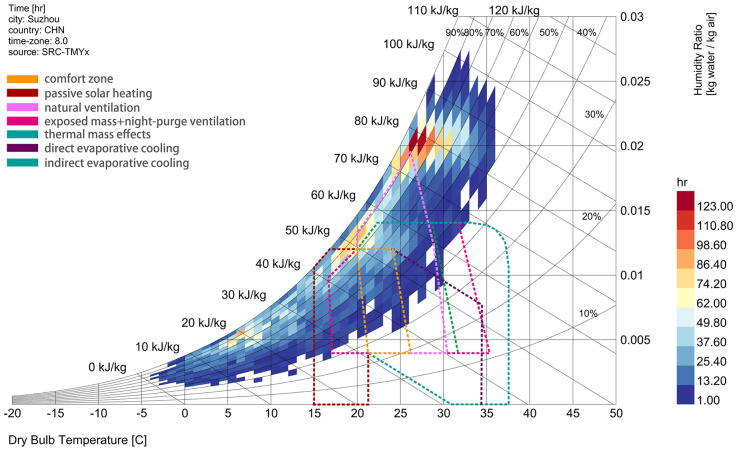
Bioclimatic charts and passive techniques for expanding thermal comfort zone.

In the first week’s bioclimatic analysis, the meteorological data of Suzhou, which features a hot humid summer and a cold wet winter, were subjected to bioclimatic analysis. Through enthalpy chart, the students found that indoor thermal and humidity conditions can be considerably improved through four passive techniques: passive solar heating, natural ventilation, thermal mass effect, and evaporative cooling (Fig 15). Among them, natural ventilation serves as the major path for the heat dissipation of buildings [59,60]. Consequently, the utilization of the “Energy Diagram” tool for wind environment simulation became an opportunity to optimize design.

The “Energy Diagram” tool served a crucial function in validating and refining design strategies within the workflow process. On the one hand, it was utilized for the meticulous verification and in-depth learning of the environmental regulation mechanisms of the reference forms, such as the “roof-patio” ventilation system from Southern Yangtze vernacular dwellings (Case II) and the “air chipper” canopy domes from Singapore South Beach Complex (Case III). On the other hand, the “Energy Diagram” tool was employed to fine-tune the design parameters of the selected forms, such as vaults and parallel walls (Case I), folded pitched roofs and solar-assisted chimneys (Case II), and an L-shaped block and double-layer curved roofs (Case III).

## Discussion

During the application of the tool, user feedback was obtained. Post-course interviews indicate that the software excels in usability and efficiency. Most students reached basic proficiency in under 10 minutes, citing an intuitive interface and streamlined workflow. Crucially, the tool supports real-time simulation with immediate feedback, enabling rapid iteration and in-class validation of design ideas.

At the workflow level, comparable studies report a CFD computation time of 20 hours per case across 29 test cases involving buildings of similar scale [55]. In contrast, the “Energy Diagram” tool requires less than 1 second of computation time. Including the time needed to draw architectural plans or sections, the average processing time per case was only 2 minutes. Previous DNN–CFD surrogate models report a per-case inference time of 350 μs for non-isothermal indoor airflow fields [57], with well-trained DNNs achieving speeds approximately 1.9 × 10⁶ times faster than CFD on a per-case basis [54]. The one-time DNN training completes within hours on a single-GPU workstation and is infrequently repeated; when adapting to new typologies, lightweight fine-tuning further limits additional training cost while preserving the real-time inference benefit. These results are consistent with our tool’s objective of providing real-time performance for LOD0–1 design stage.

Overall, the “Energy Diagram” tool significantly shortens the model–compute–visualize cycle and enhances engagement and learning outcomes. Nevertheless, there are several issues worthy of further consideration.

ⅰ. **Visual integration of environmental knowledge**

**Fig 16:**
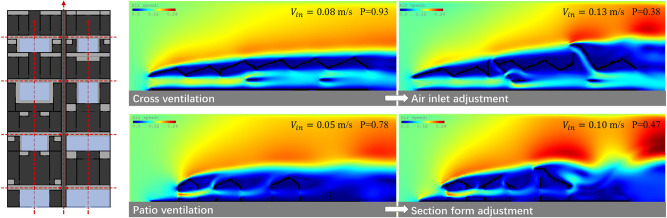
Simulation analysis on patio ventilation and cross ventilation in Southern Yangtze vernacular dwellings (Case II).

Traditional architectural pedagogy often segregates environmental physics from form-making. The “Energy Diagram” tool bridges this gap through a three-phase knowledge conversion process: observation-operation-evaluation cycle. The application cases demonstrate that users of the “Energy Diagram” tool can learn the corresponding environmental technology and knowledge from the perspective of problem solving and effectively integrate theories, strategies, and methods of sustainable environmental design into their designs. For instance, drawing inspiration from Southern Yangtze vernacular strategies (Cases II), the tool translated abstract principles like cross ventilation and patio airflow into interactive simulations. In the Mahjong Parlor project, iterative patio dimension and inlet adjustments clarified wind-induced ventilation mechanics. With inlet optimization, cross ventilation increased the indoor average wind velocity $V_{in}$ from 0.08 to 0.13 m/s, while the wind-field uniformity P (lower = more uniform) dropped from 0.93 to 0.38; with section adjustments, patio ventilation raised $V_{in}$ from 0.05 to 0.10 m/s and reduced P from 0.78 to 0.47, yielding a stronger, more uniform indoor airflow (Fig 16). This tangible workflow resolves the longstanding pedagogical challenge of tacit environmental knowledge transmission.

ⅱ. **Geometric translation of environmental logic**

**Fig 17:**
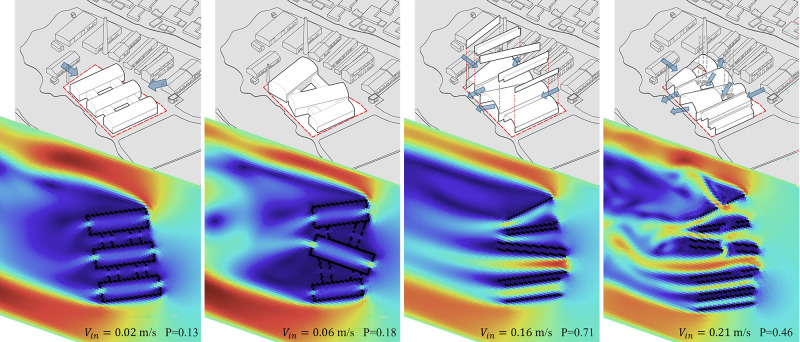
The continuous feedback and adjustment through “form-energy” interaction (Case I).

In essence, the “Energy Diagram” tool provides intuitive training that associates geometric architectural forms with environmental performance, promoting the adjustment and optimization of design parameters in the continuous “form-energy” interaction. In the Dock Museum project, the geometric parameters of the vaults, parallel walls, and patios underwent modifications via interactive simulation, ultimately aligning them with summer wind vectors (Fig 17). Iterative adjustments to the plan geometry led to progressive gains in natural ventilation, with $V_{in}$ rising from 0.02 to 0.21 m/s, culminating in P = 0.46 and a robust and acceptably uniform indoor wind field. These operations transcend typological conventions, establishing parametric relationships between geometry and environmental logic.

ⅲ. **Human-machine collaborative design situations**

**Fig 18:**
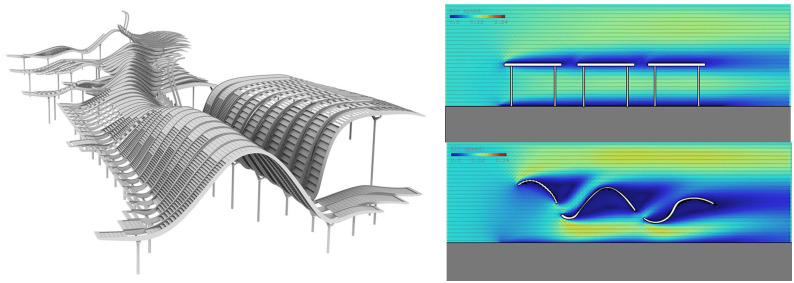
The canopy domes of the South Beach Complex in Singapore, and the simulation verification of the effectiveness “air chipper (Case III)”.

In the realm of architectural design, serendipitous innovation often arises from unexpected adaptations and creative solutions to constraints. The “Energy Diagram” tool preserves productive ambiguities that incubate innovation, is not a substitute for design judgment, but rather enhances the subjectivity of architects. The critical validation of the “air chipper” canopy domes from the Singapore South Beach Complex in the Water Market case (Fig 18) demonstrates two dimensions of tool usage: it can serve as a validation tool for existing solutions and also as a generative medium for form innovation. The human-machine collaborative design situations created by the “Energy Diagram” tool reinforce architectural agency by resisting technological determinism.

## Conclusion

This study addresses the critical gap between designers’ workflow and existing simulation tools in sustainable architectural design, particularly during the early stages of wind environment optimization. By synthesizing comparative software analysis, questionnaire survey data, and interdisciplinary thinking paradigms, the research identifies core challenges in integrating performance-driven simulation into design practice. The development and validation of the “Energy Diagram” tool demonstrate a viable pathway to bridge these gaps, offering actionable insights for advancing simulation-driven design methodologies.

### Key findings

**Disconnect Between Tools and Design Stages**: Existing simulation software predominantly caters to later design phases (LOD3–4), neglecting the formative early stages (LOD0–1) where design decisions exert the greatest impact on energy performance. The questionnaire survey highlights a stark mismatch between designers’ demand for early-stage simulation tools (62.10% desired Sketch Design integration) and the scarcity of accessible solutions (only 3.78% usage at LOD0).

**Paradigmatic Divergence**: The epistemological and methodological differences between designers and engineers—form versus energy, situational synthesis versus model isolation—create systemic barriers to tool adoption. Designers prioritize usability, visualization, and iterative feedback (60.44% cited ease of use as critical), while engineers focus on precision and computational rigor, leading to tools misaligned with design workflows.

**Tool Efficacy**: The “Energy Diagram” tool, integrating 2D zonal models and machine learning, successfully balances accuracy, efficiency, and usability for early-stage design. Validation studies show errors reduced to 16.85% against wind tunnel data, 19.21% against field measurements, and 10.45% and 13.79% against CFD simulations respectively. meeting the precision demands of conceptual design while enabling rapid iteration.

### Contributions

By coupling architectural diagrams with simplified physical models and data-driven prediction, the tool translates geometric primitives into environmental performance metrics, enabling real-time feedback during sketching. This hybrid approach resolves the “form-energy” dichotomy, empowering designers to explore climate-responsive geometries without advanced computational expertise.

The pedagogical case study illustrates how the tool fosters tacit knowledge transfer, transforming abstract environmental principles (e.g., cross-ventilation, solar chimney effects) into tangible design strategies. Students demonstrated enhanced ability to iteratively refine vaults, patios, and canopy geometries based on numerical simulations, bridging theory and practice.

The tool’s “drawing–simulation–optimization” workflow reduces reliance on post-hoc performance validation, instead positioning environmental logic as a generative driver of form. This aligns with designers’ preference for visual, iterative processes while maintaining scientific rigor through ML-enhanced predictions.

### Limitations and future work

The questionnaire survey focused on China-based users, where prevailing climatic and regulatory contexts make natural ventilation a salient design goal; results may not generalize to regions with different climates, codes, or design cultures. Voluntary online recruitment and the respondent mix may introduce self-selection and role bias.

While the “Energy Diagram” tool shows promise, limitations persist. Firstly, current 2D simulations may oversimplify 3D wind behaviors, particularly for complex geometries like curved roofs or staggered volumes. Future iterations could incorporate 2.5D layered models or lightweight 3D Lattice–Boltzmann methods. Secondly, the DNN model’s accuracy relies on training data from specific climatic contexts. Expanding the dataset to diverse climates and building typologies would enhance its generalizability. Thirdly, as a standalone platform, the tool faces compatibility challenges with mainstream BIM/CAD software. Developing plugins for Rhino/Grasshopper or Revit could broaden its adoption.

### Final statement

This research underscores the necessity of human-centric tool design in sustainable architecture. By prioritizing designers’ cognitive patterns and workflow needs, the “Energy Diagram” exemplifies how digital tools can democratize performance simulation without oversimplifying scientific principles. Future advancements in AI-assisted design, coupled with interdisciplinary collaboration frameworks, hold potential to further harmonize creativity and sustainability in the built environment.

The transition to low-carbon architecture demands tools that empower designers as proactive agents of environmental innovation. The “Energy Diagram” represents a critical step toward this vision, transforming wind environment simulation from a specialist task into an intuitive, integral component of the design process. As digital technologies evolve, such tools will increasingly blur the lines between form-making and performance optimization, fostering architectures that are as responsive to climate as they are to human aspiration.

## Supporting information

S1 File Original questionnaire. (DOCX)

S2 File Translated questionnaire. (DOCX)

S3 File Questionnaire database. (XLSX)
