# Supplementary material for: Energy diagram: Investigation and application of a design-thinking-driven wind environment simulation tool for sustainable architecture
Source: PLoS One. 2026 Feb 11;21(2):e0342247. doi: 10.1371/journal.pone.0342247 (PMC12893547; doi:10.1371/journal.pone.0342247)
Supplement: S1 File — (DOCX) [file pone.0342247.s001.docx]

建筑设计行业风环境模拟软件使用情况问卷调查

1. 您的职业状态是？

| - 从业人员 | - 学生 |
| --- | --- |

1. 您所在的设计行业领域是？

| - 建筑设计 | - 城乡规划 | - 风景园林 | - 环境工程 |
| --- | --- | --- | --- |

1. 您是否在设计过程中使用过风环境模拟软件？

| - 是 |
| --- |
| - 否（如果选择“否”，请直接跳至第9题） |

1. 您最常用的风环境模拟软件是什么？（可多选）

| - OpenFOAM |
| --- |
| - Ansys Fluent |
| - Phoenics |
| - Butterfly |
| - Envi-met |
| - Winair |
| - SimScale |
| - EnergyPlus/OpenStudio |
| - DesignBuilder |
| - TRNSYS |
| - eQUEST |

1. 您选择当前软件的主要原因是什么？（可多选）

| - 软件易于学习和使用 |
| --- |
| - 软件功能强大，满足设计需求 |
| - 软件计算速度快，效率高 |
| - 软件后处理能力强，结果可视化效果好 |
| - 软件GUI界面友好，操作便捷 |
| - 软件平台兼容性好，易于集成到其他系统中 |
| - 其他（请具体说明）：__________________ |

1. 您最常在设计的哪个阶段进行风环境模拟？（可多选）

| - 草图设计 |
| --- |
| - 方案设计 |
| - 初步设计 |
| - 扩初设计 |
| - 施工图设计 |

1. 您在项目中进行风环境模拟与设计交互反馈的平均循环次数是？

| - 1-2次 |
| --- |
| - 3-5次 |
| - 5-10次 |
| - 10次以上 |

1. 在使用风环境模拟软件的过程中，您遇到过哪些挑战或问题？（可多选）

| - 软件操作复杂，难以掌握 |
| --- |
| - 软件计算结果不准确，需要反复调整 |
| - 计算成本高，影响设计效率 |
| - 软件后处理功能不足，难以得到满意的可视化结果 |
| - 软件GUI界面不友好，操作不便 |
| - 软件平台兼容性差，与其他系统难以集成 |
| - 其他（请具体说明）：__________________ |

1. 您认为理想的风环境模拟软件有哪些功能是必不可少的？（限选三项）

| - 易于学习和使用 |
| --- |
| - 精确的计算结果 - 高效反馈 |
| - 强大的后处理功能，包括可视化结果及其分析 |
| - 用户友好的GUI界面 |
| - 良好的平台兼容性 |
| - 其他（请具体说明）：__________________ |

1. 您是否希望在草图阶段进行风环境模拟?

| - 是 |
| --- |
| - 否   理由是：__________________ |
