# Supplementary material for: Energy diagram: Investigation and application of a design-thinking-driven wind environment simulation tool for sustainable architecture
Source: PLoS One. 2026 Feb 11;21(2):e0342247. doi: 10.1371/journal.pone.0342247 (PMC12893547; doi:10.1371/journal.pone.0342247)
Supplement: S2 File — (DOCX) [file pone.0342247.s002.docx]

Translated English version of the questionnaire

1. **What is your professional status?**

| - Practitioner | - Non-resident |
| --- | --- |

1. **Which design industry sector do you work in?**

| - Architectural Design (envelope-, building-, and district-scale) |
| --- |
| - Urban and Rural Planning (district-, and city-scale) |
| - Landscape Design |
| - Environmental Engineering |

1. **Have you used wind environment simulation software in your design process?**

| - Yes |
| --- |
| - No (If "No," please skip to Question 9) |

1. **Which wind environment simulation software do you use most frequently? (Multiple choices allowed)**

| - OpenFOAM | - SimScale |
| --- | --- |
| - Ansys Fluent | - EnergyPlus/OpenStudio |
| - Phoenics | - DesignBuilder |
| - Butterfly | - TRNSYS |
| - Envi-met | - eQUEST |
| - Winair |  |

1. **What are the main reasons for your choice of current software? (Multiple choices allowed)**

| - The software has a user-friendly GUI interface and is easy to learn and use |
| --- |
| - The software is high-accuracy and meets design requirements |
| - The software has fast calculation speeds and high efficiency |
| - The software has strong post-processing capabilities and good visualization results |
| - The software has good platform compatibility and is easy to integrate into other systems |
| - Other (Please specify): __________________ |

1. **At which stage of design do you most frequently perform wind environment simulation?**

| - Sketch Design |
| --- |
| - Scheme Design |
| - Project Design |
| - Extended Preliminary Design |
| - Construction Documents Design |

1. **What is the average number of cycles of wind environment simulation and design interaction feedback you conduct in a project?**

| - 1-2 times |
| --- |
| - 3-5 times |
| - 6-10 times |
| - 11-20 times |

1. **What challenges or issues have you encountered while using wind environment simulation software? (Multiple choices allowed)**

| - The software's GUI interface is not user-friendly and difficult to master |
| --- |
| - The software's calculation results are inaccurate and require repeated adjustments |
| - The calculation costs are high, affecting design efficiency |
| - The software lacks sufficient post-processing capabilities, making it difficult to obtain satisfactory visualization results |
| - The software has poor platform compatibility and is difficult to integrate with other systems |
| - Other (Please specify): __________________ |

1. **What are the three essential features of an ideal wind environment simulation software in your opinion? (Select up to three)**

| - User-friendly GUI interface and Easy to learn and use |
| --- |
| - Accurate calculation results |
| - Efficient feedback |
| - Powerful post-processing capabilities, including visualization and analysis of results |
| - Good platform compatibility |
| - Other (Please specify): __________________ |

1. **Do you wish to perform wind environment simulation at the sketch design stage?**

| - Yes |
| --- |
| - NO |
| Reason: __________________ |
